# Machine learning and atherosclerotic cardiovascular disease risk prediction in a multi-ethnic population

**DOI:** 10.1038/s41746-020-00331-1

**Published:** 2020-09-23

**Authors:** Andrew Ward, Ashish Sarraju, Sukyung Chung, Jiang Li, Robert Harrington, Paul Heidenreich, Latha Palaniappan, David Scheinker, Fatima Rodriguez

**Affiliations:** 1 Department of Electrical Engineering, Stanford University, Stanford, CA USA; 2 Division of Cardiovascular Medicine, Stanford University School of Medicine, Stanford, CA USA; 3 Palo Alto Medical Foundation Research Institute, Palo Alto, CA USA; 4 Division of Primary Care and Population Health, Stanford University School of Medicine, Stanford, CA USA; 5 Department of Management Science and Engineering, Stanford University School of Engineering, Stanford, CA USA; 6 Division of Pediatric Endocrinology and Diabetes, Stanford University School of Medicine, Stanford, CA USA

**Keywords:** Cardiovascular diseases, Epidemiology

## Abstract

The pooled cohort equations (PCE) predict atherosclerotic cardiovascular disease (ASCVD) risk in patients with characteristics within prespecified ranges and has uncertain performance among Asians or Hispanics. It is unknown if machine learning (ML) models can improve ASCVD risk prediction across broader diverse, real-world populations. We developed ML models for ASCVD risk prediction for multi-ethnic patients using an electronic health record (EHR) database from Northern California. Our cohort included patients aged 18 years or older with no prior CVD and not on statins at baseline (*n* = 262,923), stratified by PCE-eligible (*n* = 131,721) or PCE-ineligible patients based on missing or out-of-range variables. We trained ML models [logistic regression with L_2_ penalty and L_1_ lasso penalty, random forest, gradient boosting machine (GBM), extreme gradient boosting] and determined 5-year ASCVD risk prediction, including with and without incorporation of additional EHR variables, and in Asian and Hispanic subgroups. A total of 4309 patients had ASCVD events, with 2077 in PCE-ineligible patients. GBM performance in the full cohort, including PCE-ineligible patients (area under receiver-operating characteristic curve (AUC) 0.835, 95% confidence interval (CI): 0.825–0.846), was significantly better than that of the PCE in the PCE-eligible cohort (AUC 0.775, 95% CI: 0.755–0.794). Among patients aged 40–79, GBM performed similarly before (AUC 0.784, 95% CI: 0.759–0.808) and after (AUC 0.790, 95% CI: 0.765–0.814) incorporating additional EHR data. Overall, ML models achieved comparable or improved performance compared to the PCE while allowing risk discrimination in a larger group of patients including PCE-ineligible patients. EHR-trained ML models may help bridge important gaps in ASCVD risk prediction.

## Introduction

Risk assessment algorithms have a well-established role in guiding atherosclerotic cardiovascular disease (ASCVD) management. The 2018 update to the 2013 ACC/AHA prevention guidelines highlighted the use of the revised pooled cohort equations (PCE) to determine ASCVD risk, which is used to guide crucial management decisions, particularly the initiation of moderate to high-intensity statin therapy for long-term risk reduction^1^. Developed using Cox proportional hazards modeling, the PCE represent a widely used guideline-endorsed calculator to assess 10-year ASCVD event risk for individuals without a prior history of ASCVD, and are a central part of ASCVD risk reduction approaches in clinical settings^2^. For calculating an individualized 10-year risk of ASCVD, the PCE are applicable to patients with characteristics within prespecified ranges—including age, race, cholesterol levels, and systolic blood pressure. PCE use is consequently limited for patients with values outside the prespecified PCE ranges as well as patients without all variables available, preventing accurate guideline-based risk estimation to guide ASCVD risk reduction strategies. The PCEs were derived primarily from non-Hispanic Whites (NHWs) and African-American (AA) populations, and it is not clear whether they adequately identify risk in diverse populations based on recent analyses demonstrating inconsistent results^3^. Indeed, the ACC/AHA Work Group that developed the PCE noted that the risk estimator may not accurately predict risk in other racial/ethnic groups^4^.

Machine learning (ML) algorithms present an opportunity to develop improved and more generalizable risk prediction models. By leveraging large-scale data—from electronic health records (EHRs), for instance—such algorithms can determine combinations of variables that reliably predict an outcome. ML may incorporate variables that may not be otherwise considered in traditional predictive algorithms and thus demonstrate favorable performance compared to traditional methods^5^. Predictive ML algorithms have been applied broadly in various disciplines^6^, with burgeoning evidence of their potential utility in medicine^7–10^.

The applicability of the PCE to patients with missing or out-of-range PCE variables or in racial/ethnic subgroups other than NHW or AAs is limited. The aim of this study was to develop EHR-trained ML-based ASCVD risk prediction algorithms that demonstrate broad real-world applicability, including random forests (RF), gradient boosted machines (GBM), extreme gradient boosted models (XGBoost), and logistic regression with the standard L_2_ penalty (LRL2), and with an L_1_ lasso penalty (LRLasso). We hypothesized that ML methods would allow the development of ASCVD risk prediction algorithms that perform favorably compared to PCE and allow risk estimation for patients to whom the PCE do not apply.

## Results

### Cohort selection and baseline characteristics

**Fig. 1:**
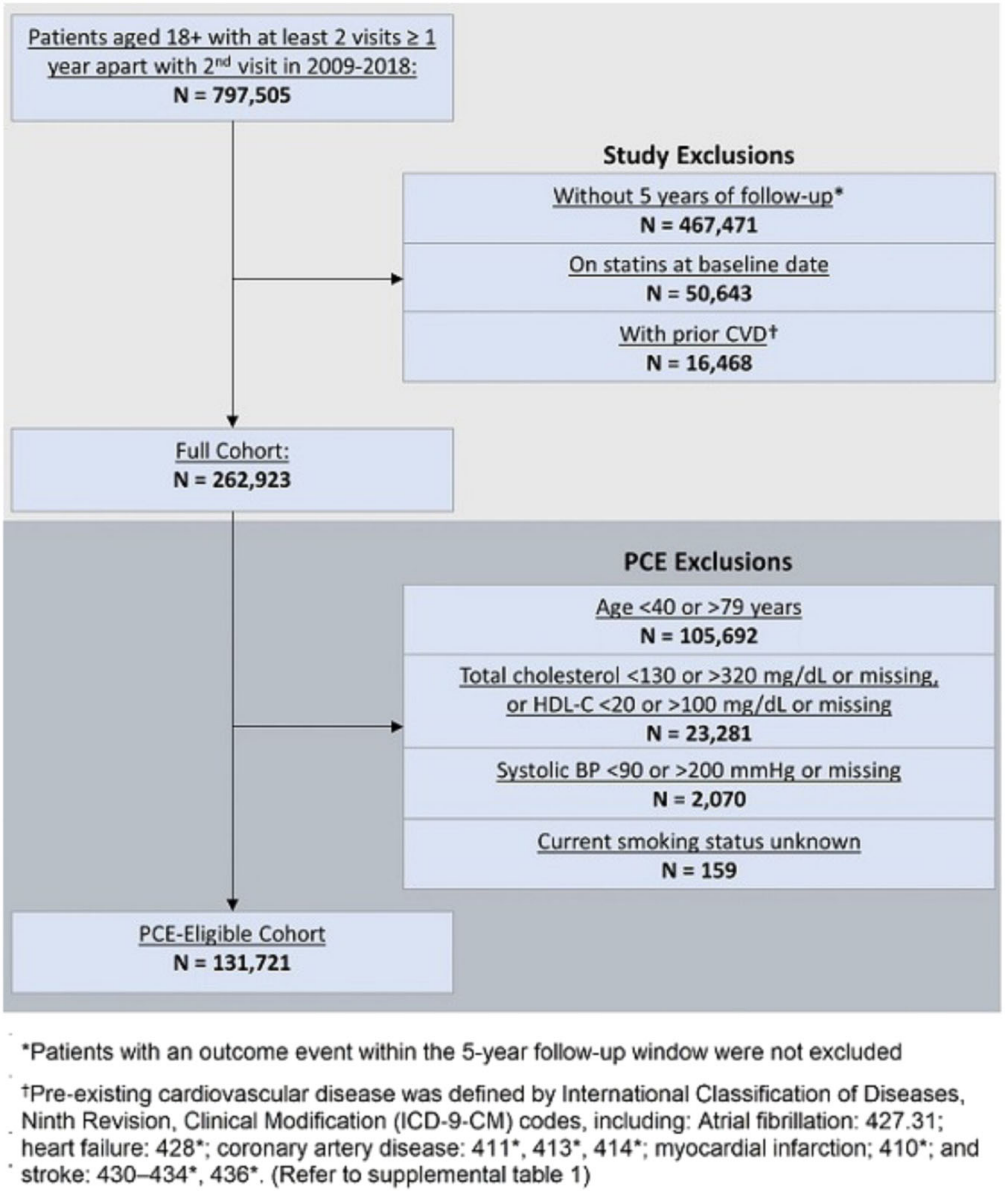
Consort diagram. N number, ASCVD atherosclerotic cardiovascular disease, CVD cardiovascular disease, PCE pooled cohort equation, HDL-C high-density lipoprotein cholesterol, BP blood pressure. *Patients with an outcome event within the 5-year follow-up window were not excluded. ^†^Pre-existing cardiovascular disease was defined by International Classification of Diseases, 9th revision, Clinical Modification (ICD-9-CM) codes, including: atrial fibrillation: 427.31; heart failure: 428*; coronary artery disease: 411*, 413*, 414*; myocardial infarction: 410*; and stroke: 430–434*, 436* (refer to Supplementary Table 1).

Of 797,505 patients, 518,114 patients using statins or with prior CVD at baseline, or with <5 years of ASCVD event-free follow-up after their index visit, were excluded. The final study population (the “full cohort”) included 262,923 patients with 4309 ASCVD-related events during 5-year follow-up. Within the full cohort, 105,692 patients were not aged 40–79 years on their baseline date and 25,510 patients’ cholesterol, blood pressure, or smoking status were unknown or out of range. This left 131,721 PCE-eligible patients, and 2232 with an ASCVD event during follow-up (Fig. 1).

Characteristics of the PCE-eligible cohort and the full cohort are shown in Table 1. The full cohort included 49% NHWs, 30% Asians, and 8% Hispanics. The average patient age was 45.3 years, and 59% were females. Racial/ethnic demographics of PCE-eligible patients were qualitatively similar to the full cohort, as were relevant cardiovascular comorbidities, including systolic blood pressure, lipid levels, and proportion of current smokers and those with type 2 diabetes. There were 1175 EHR variables that were created and considered along with the nine PCE variables: diastolic BP, height, weight, 559 medication boolean values (by GPI4), 146 boolean lab values, and 2 “# of lab values,” 279 CCS diagnosis class boolean variables, 156 family history variables, 3 medical utilization variables (number of primary, specialty, and other care visits), and 7 socioeconomic variables (median household income, and variables representing six levels of education). Supplementary Data 1 shows the additional variables selected for the PCE+ variable set. The level of missingness of each PCE variable, as well as additional continuous variables considered, is shown in Supplementary Table 1.

**Table 1:** Baseline characteristics of the full cohort and PCE-eligible cohort.

	Full cohort	PCE-eligible cohort
*N*	262,923	131,721
Age, mean (SD)	45.38 (14.9)	52.63 (9.4)
Female (%)	154,569 (58.8)	74,666 (56.7)
Race/ethnicity (%)		
Non-Hispanic White	127,639 (48.6)	72,415 (55.0)
Asian	78,553 (29.9)	34,555 (26.2)
Hispanic	21,003 (8.0)	8,999 (6.8)
African American	3945 (1.5)	2127 (1.6)
Other race	4758 (1.8)	2051 (1.6)
Missing race	27,025 (10.3)	11,574 (8.8)
SBP (mmHg) mean (SD)	119.59 (16.2)	122.04 (16.1)
On antihypertensive medication (%)	34,490 (13.1)	23,713 (18.0)
HDL cholesterol (mg/dL), mean (SD)	55.24 (16.0)	56.02 (15.0)
Total cholesterol (mg/dL), mean (SD)	190.45 (35.6)	196.64 (32.7)
Current smoker (%)	13,055 (5.0)	5807 (4.4)
Type 2 diabetes (%)	10,209 (3.9)	5690 (4.3)
Estimated 5-year ASCVD risk (%)	NA	2.0
Estimated 10-year ASCVD risk (%)	NA	4.9

*PCE* pooled cohort equations, *N* number, *SBP* systolic blood pressure, *SD* standard deviation, *HDL* high-density lipoprotein, *NA* not applicable.

### ML model and PCE performance

**Fig. 2:**
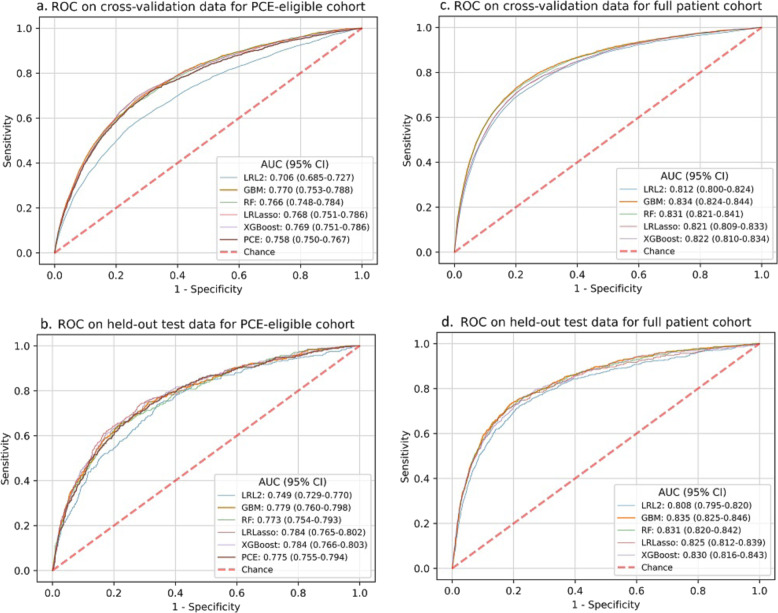
ROC curves for PCE and ML model performance. ROC curves are outlined for PCE versus model ML performance in the PCE-eligible cohort (in **a** cross-validation and **b** held-out test data) and ML model performance on the full cohort (in **c** cross-validation and **d** held-out test data). Legend entries denote the AUC for each method with 95% confidence intervals. ROC receiver-operating characteristic, PCE pooled cohort equations, ML machine learning, AUC area under receiver-operating characteristic curve, LRL2 logistic regression with an L_2_ penalty, LRLasso logistic regression with an L_1_ (lasso) penalty, RF random forest, GBM gradient boosting machine, XGBoost extreme gradient boosting.

Figure 2 shows the receiver-operating characteristic (ROC) curves of the best-performing cross-validated LRL2, LRLasso, RF, GBM, and XGBoost models in the validation and test sets from the PCE-eligible cohort and the full cohort. PCE performance was assessed in the PCE-eligible cohort. Most ML models performed comparably or better than the PCE (area under receiver-operating characteristic curve (AUC) 0.758) in the validation set of the PCE-eligible cohort (Supplementary Table 2). In the corresponding test set, ML models demonstrated performance similar to the PCE (AUC 0.775, 95% confidence interval (CI): 0.755–0.794). In the full cohort including patients ineligible for PCE application due to missing/out-of-range data, GBM was the best-performing model in the test set (AUC 0.835, 95% CI: 0.825–0.846), with better risk discrimination compared to that of the PCE (AUC 0.775, 95% CI: 0.755–0.794) in PCE-eligible test patients. GBM in the full cohort demonstrated higher sensitivity (lower false-negative rate) compared to that of the PCE in PCE-eligible patients at prevalence thresholds of 5, 10, or 25% high-risk patients (Supplementary Table 3).

On Hispanic and Asian patients in the test set, LRLasso achieved overall risk discrimination comparable to the PCE (Table 2). There was no substantial improvement in ML model performance after incorporation of additional EHR variables (PCE+) compared with PCE-only variables in these subgroups. Table 3 shows the performance of the best-performing ML model (GBM) across all patients aged 40–79 years, including those with missing or out-of-range PCE variables, while considering only PCE variables or incorporating additional EHR variables (PCE + ). GBM was the best-performing model in cross-validation using both variable sets (Supplementary Table 2), and achieved similar performance on the test set when trained with the PCE + variables (AUC 0.790, 95% CI: 0.765–0.814) than when trained only with the PCE variables (AUC 0.784, 95% CI: 0.759–0.808). Oversampling did not substantially affect GBM performance with PCE-only variables or with additional EHR variables beyond PCE (PCE + ). Predictive variables identified by ML models beyond traditional PCE parameters included socioeconomic factors (Supplementary Table 4). Iterative imputation outperformed mean-value imputation methods (Supplementary Table 5). When imputation was used among patients aged 40–79 with missing PCE variables, GBM performance was similar to that of the PCE (Supplementary Table 5). ML model performance in Asian and Hispanic groups after being trained on NHW and AA populations only is also reported (Supplementary Table 6).

**Table 2:** AUCs for Asian and Hispanic patients without missing PCE variables.

Race	*N*	Variables	LRLasso AUC (95% CI)	PCE AUC (95% CI)
Asian	34,555	PCE	0.804 (0.744–0.864)	**0.807 (0.748–0.866)**
Asian	34,555	PCE+	0.803 (0.743–0.863)	**0.807 (0.748–0.866)**
Hispanic	8999	PCE	**0.752 (0.645–0.859)**	0.742 (0.634–0.851)
Hispanic	8999	PCE+	**0.768 (0.663–0.874)**	0.742 (0.634–0.851)

The model with the highest test AUC for each subgroup is given in bold.

*AUC* area under receiver-operating characteristic curve, *N* number, *PCE* pooled cohort equation, *PCE+* pooled cohort equation variables with additional electronic health record variables, *LRLasso* logistic regression with an L1 (lasso) penalty.

**Table 3:** AUCs for patients aged 40–79 years including those who were missing one or more PCE variables.

*N*	Variables	GBM AUC (95% CI)	PCE AUC (95% CI)
157,231	PCE	**0.784 (0.759–0.808)**	0.774 (0.757–0.790)
157,231	PCE+	**0.790 (0.765–0.814)**	0.774 (0.757–0.790)

The model with the highest AUC for each subgroup is given in bold.

*AUC* area under receiver-operating characteristic curve, *N* number, *PCE* pooled cohort equation, *PCE+* pooled cohort equation variables with additional electronic health record variables, *GBM* gradient boosting machine.

## Discussion

In a large, diverse population of patients from an EHR-based cohort, we found that ASCVD risk prediction ML models classify significantly more patients than the PCE with comparable or improved performance. These models increase the clinical applicability of ASCVD risk prediction models by including patients with missing or invalid PCE variables and Asian and Hispanic populations, which were not considered in PCE derivation cohorts. We also found that incorporating structured EHR data beyond PCE variables did not substantially improve ML model risk discrimination.

PCE incorporate several cardiovascular risk predictors to estimate an individual’s 10-year risk of ASCVD and guide treatment decisions such as statin initiation, blood pressure goals, and aspirin use. For patients with missing PCE data or ethnic/racial populations not included in the PCE derivation cohort, management decisions may be affected by risk over- or underestimation. In a large cohort of Kaiser Permanente Northern California patients, the PCE overestimated ASCVD across diverse groups^11^. In an outpatient population from Northern California, PCE-based risk estimation demonstrated heterogeneity among diverse groups, including AAs, Asians, and Hispanics, with significantly lower degree of risk overestimation compared to the Kaiser population^3^.

While the PCE is the ACC/AHA recommended ASCVD risk prediction tool by contemporary guidelines, alternative CVD risk prediction tools derived from US populations include the Framingham General CVD risk profile and the Reynolds Risk Score. Based on its derivation cohort of largely white individuals, however, the Framingham risk profile has uncertain performance among other racial/ethnic groups. The Reynolds Risk Score incorporates sex-specific equations for ASCVD risk prediction; however, it was derived from largely white individuals enrolled in clinical trials, with uncertain performance in other racial/ethnic groups. The QRISK score developed in Great Britain and the European Systematic Coronary Risk Evaluation (SCORE) algorithm may be less applicable in the United States given their derivation cohorts^12^.

Several groups have reported PCE recalibration to optimize risk prediction. A study from the Women’s Health Initiative demonstrated that a statistical model incorporating events from the Centers for Medicare and Medicaid Services allowed improved risk discrimination^13^. This study did not address patients with missing variables or racial/ethnic groups other than NHW and AA patients. A study that recalibrated PCE using modern cohort data with improved risk prediction did not address effects on diverse racial ethnic/racial subgroups^2^. To our knowledge, our study is the first to explore ML-derived models for ASCVD risk prediction in patients with missing PCE variables and diverse racial/ethnic subgroups.

Our results have implications for ASCVD risk prediction. As a tree-based ML method, GBM is able to incorporate complex variable interactions, which may explain its favorable performance compared to non-tree-based models, such as the PCE, as has been demonstrated in other contemporary ML studies addressing cardiovascular disease^14^. Our algorithm was more broadly applicable compared to PCE, including for individuals with missing variables or values outside prespecified ranges—which may be important in practice. In our study, 48% of ASCVD events occurred in patients ineligible for PCE application. In a study of Northern California patients, out of 941,546 patients with available lipid levels, ~25% (232,497) had missing or out-of-range PCE values and were excluded from traditional PCE application^11^. ML-based models may help bridge ASCVD risk prediction gaps with such patients to allow guideline-directed management based on risk assessment. In addition, we found that GBM performance in the full cohort had favorable sensitivity compared to that of the PCE in PCE-eligible patients when assuming intermediate prevalence of high-risk patients of 5, 10, or 25%, which further supports the clinical utility of using ML models. For an initial screening test for ASCVD risk, sensitivity (associated with false-negative rate) is a crucial performance metric in order to minimize misclassifying high-risk patients as low risk, and maximize opportunities to initiate potentially life-saving management.

Consistent with other studies, we found that incorporating structured EHR data beyond traditional PCE variables in ML models did not substantially improve risk prediction. While we did not check inter-correlation of EHR variables, it is likely that we used correlated variables, which we believe may be helpful for ML methods when variables are missing for a patient. The top 20 predictive variables employed by ML models included traditional variables, such as lipids along with socioeconomic factors, including education level. A study from the UK Biobank found that the addition of apolipoproteins, direct, or calculated low-density lipoprotein (LDL) cholesterol did not improve ASCVD risk prediction^15^. This analysis did not assess variables beyond lipid levels. Our results raise the hypothesis that efforts at improving ASCVD risk prediction by capturing additional EHR variables may be limited. A previously reported ML-based risk calculator derived from the Multi-Ethnic Study of Atherosclerosis used the same variables as PCE^16^. These findings suggest that the development of improved risk prediction may require richer data or incorporation of novel variables, such as genetic information across larger patient groups, although incorporation of genetic information may be susceptible to additional bias if not carefully performed^17,18^.

PCE performance and prevalence of ASCVD risk factors vary across racial/ethnic subgroups^3^. We found that ML-based risk prediction was comparable to PCE in Asians and Hispanics, and that incorporation of additional EHR variables did not substantially improve risk discrimination. Novel ASCVD risk estimation models aimed at improved performance in racial/ethnic subgroups may require consideration of variables that are not adequately captured in current structured EHRs.

Our study has additional strengths. We report mean AUCs from cross-validation and AUCs from test set results for the ML algorithms, with the held-out test set separated at the beginning. Our population was enriched for Asian and Hispanic subgroups, allowing us to explore risk prediction in understudied populations. We had a significant number of ASCVD events comparable to similar studies^11^. We report the effect of oversampling on ML model performance (Supplementary Table 7) as well as both mean AUCs from cross-validation and AUCs from test data with iterative imputation and mean-value imputation (Supplementary Table 5).

Our study should be interpreted in the context of its limitations. The study population consisted of diverse, likely insured patients from a multipayer private health system in Northern California and may not be generalizable across the United States. We used 5-year ASCVD risk rather than 10-year risk as typically reported by PCE, which raises the possibility of missing later events. However, this approach has been used previously in comparable studies, with observation of a linear ASCVD risk during the first 5 years^11,19^. Due to limitations in computational power available for the analysis of protected health information-sensitive data, we performed feature selection, hyperparameter tuning, and oversampling rate selection using separate cross-validation experiments within our model selection pipeline, which could potentially result in suboptimal performance. We performed model assessment on a held-out 20% test set separate from our cross-validation sets to minimize bias in assessment. Performing nested cross-validation could potentially minimize bias in model comparison and assessment further. We did not disaggregate Asian and Hispanic patients, which may mask heterogeneity within these subgroups^3^. We employed EHR and Social Security records data, which have limitations inherent to systems not built for research purposes. For example, as previously noted, race/ethnicity data were largely self-reported and otherwise inferred by validated methods, which may contribute to misclassification^3^.

In conclusion, we evaluated the performance of ML-based models for ASCVD risk prediction compared to PCE in a large, diverse population from Northern California. We found the ML-based models performed comparably or better than PCE while allowing risk discrimination in a substantially larger group of patients, including those with missing or invalid PCE imputation variables, which may be relevant to practice. Incorporation of additional EHR data beyond traditional PCE variables did not substantially improve ML model performance, which may have implications for efforts aimed at improving risk prediction using structured EHR data. We found no decline in ML-based model performance compared to PCE in Asian and Hispanic subgroups. Our results inform the potential utility of ML-based models to bridge important gaps in clinical ASCVD risk prediction through broader applicability.

## Methods

### Study sample

The study was approved by the Stanford University Institutional Review Board (IRB), who determined that the research does not involve human subjects and granted a waiver of consent based on the nature of the project, including the use of previously collected, de-identified data. The study sample was selected from EHR data of adults >18 years of age in a community-based outpatient healthcare system in Northern California between January 1, 2009 and December 31, 2018 with at least two outpatient visits that were at least 1 year apart. The index date was defined as the first outpatient visit, which was 1 year after the patient’s first clinic visit. If there were no cholesterol lab results before a patient’s index date, the index date was shifted to the date of the first cholesterol lab result. Patients with pre-existing ASCVD, atrial fibrillation, heart failure, or other cardiovascular disease identified by the International Classification of Diseases, 9th and 10th revision (ICD-9-CM/ICD-10-CM) coding scheme were excluded (Supplementary Table 8). Patients on statins at baseline dates, identified by the first two digits of generic product identifier (GPI2) codes 39, were excluded. Patients were stratified by whether they were PCE eligible or ineligible. PCE-eligible patients were those for whom all variables used in the PCE were available and within prespecified ranges, as described below.

### Demographic and clinical variables

Patient variables used by the PCE include sex, age, race, total and high-density lipoprotein (HDL) cholesterol, systolic blood pressure, blood pressure treatment status, smoking status, and diabetes status; we refer to these as the PCE variables. For each patient, for total cholesterol, HDL cholesterol, systolic blood pressure, and smoking status, the most recent value on or before the index date was used. On the date of the patient’s blood pressure measurement, the use of antihypertensive medications (GPI codes beginning with 33, 34, 36, 37, 4013, or 4016) was assessed. Diabetes status was identified by either a diagnosis of diabetes (ICD-9-CM: 250.*; ICD-10-CM: E11*, Z79.4, Z79.84) or a diabetes medication (GPI2: 27) prescribed on or prior to index date. Patients were included in the PCE-eligible cohort if all PCE variables were present and within specified ranges: age 40–79 years, total cholesterol 130–320 mg/dL, HDL-C 20–100 mg/dL, and systolic blood pressure 90–200 mmHg. Race/ethnicity was self-reported. Some patients’ races were inferred based on the Social Security Record database as previously described^20^.

### Additional EHR variables

We assessed the value of including additional EHR variables beyond those from the PCE. These variables were selected a priori based on EHR availability, relevance to ASCVD risk, and predictive potential. Variables were grouped into socioeconomic, clinical, and healthcare utilization categories and extracted 1 year prior to the index date.

Socioeconomic variables were based on addresses and included census block group level indicators of educational attainment and median household income.

Patient medical problems were extracted from the EHR problem list, which were coded in ICD-9-CM/ICD-10-CM, and grouped into 283 categories using the Clinical Classification Software (CCS)^21^. Self-reported family (parents or sibling) medical histories were extracted. The existence of each CCS and family history was coded as a binary variable. The most recent height, weight, and diastolic blood pressure measurements before each patient’s index date were included.

Medication prescription information was incorporated by considering the first four digits of prescriptions’ GPI codes (GPI4). An indicator was created for each GPI4 denoting whether any medication from that GPI4 was prescribed. The total number of medication prescriptions in the prior year was included as a variable.

Two indicator variables were included for each lab test: whether the test was ordered and returned a “normal” result, and whether it was ordered and returned an “abnormal” result. The total number of laboratory tests ordered and the total number which returned “abnormal” results were also included. LDL cholesterol was included if available.

Indicators of healthcare utilization included the number of primary care, urgent care, specialty, and other (e.g., ancillary, educational) services care visits in the previous year.

### Outcome

In accordance with ACC/AHA Work Group guidelines, an ASCVD event was defined as the first acute myocardial infarction, stroke, or fatal coronary artery disease^4^. Acute myocardial infarction was defined by ICD-9-CM codes 410.* and ICD-10-CM codes I21.*, I22.*, I23.3, I24.0, I24.9, I25.9, or I51.3^12,13^. Stroke events were defined based on ICD-9-CM codes 430.*, 431.*, 432.*, 433.*1, 434.*1, or 436.0 and ICD-10-CM codes G46.*, I63.*, I67.85, I69.30, I77.89, P91.0, or Z86.73^14^. Fatal coronary artery disease was defined by the presence of an ICD-9-CM code 411.*, 413.*, or 414.* or an ICD-10-CM code I20.*, I23.7, I24.*, I25.*, or T82.85 code followed by death within a year. Death information was retrieved from EHRs and Social Security records.

### Statistical analysis and ML model training

The 5-year ASCVD predicted risk was calculated for the PCE-eligible cohort using published parameter estimates from the PCE^4,22^. Parameters developed for NHWs were used to estimate values for Asian, Hispanic, and other non-AA populations.

In order to determine ML risk prediction performance, the PCE-eligible patient cohort was split into an 80% training set and a 20% test set stratified by outcome. Several ML algorithms were trained on the training set, including RF, GBM, XGBoost, and LRL2 and with an LRLasso. These were the only algorithms used. They were chosen for their ability to effectively incorporate many variables into the models returned by the algorithm. The tree-based models [RF, GBM, and XGBoost] can model complex, high-order interactions between the input variables. Analysis was performed in Python 3.7 using the scikit-learn and xgboost packages, versions 0.21.2 and 0.90, respectively^23^.

First, the ML algorithms were given the PCE variables as inputs and tasked with predicting whether a patient would have an ASCVD event in the next 5 years. For each ML algorithm, 5-fold cross-validation was used on the training set to tune hyperparameters, such as tree depth, learning rate, and number of trees. Hyperparameters used and values for those hyperparameters are shown in Supplementary Table 9. Hyperparameters were tuned to control the models’ complexities and prevent overfitting. A grid search was used for hyperparameter tuning. Models were compared based on the mean AUC, also known as the *C*-statistic, across the 5-folds. AUC CIs were calculated^24^. Once hyperparameters were selected and the best-performing cross-validated model was retrained on the entire training set, the final metrics of the best-performing ML models as well as the metrics of the PCE, including AUC, sensitivity, specificity, precision, and *F*1-score were reported and evaluated on the 20% held-out test set.

Next, ML algorithms were given access to additional variables extracted from the EHR alongside the PCE variables. As before, hyperparameters were tuned on the training set using 5-fold cross-validation. Since some EHR variables were not expected to have robust predictive power, a composite score incorporating the feature importance metrics returned by the tree-based methods as well as nonzero coefficients of LRLasso was used to prune variables (see Supplementary Note 1 for more details). The variables used by the models at the end of this pruning process are denoted as the PCE+ variables. With these PCE+ variables as inputs, hyperparameters were tuned using 5-fold cross-validation, the best model for each algorithm was retrained on the entire training set and the AUC was reported on the 20% held-out test set.

Third, ML algorithm performance for ASCVD risk prediction was assessed in the full patient cohort, which included patients for which the PCE were not designed (e.g., patients with missing or out-of-range variables, Asian/Hispanic patients). First, the hyperparameter grid search was run and ML models were trained on Asian and Hispanic subgroups, and ML performance, using only the PCE variables and using the PCE+ variables, was compared to that of the PCE. Because the number of patients in these subgroups was lower, the LRLasso model was compared to the PCE on the test set, as it is robust to overfitting when working with smaller datasets^25^. Second, patients whose PCE variables were not within prespecified ranges, as well as patients who were missing PCE variables, were incorporated in the ML model training process. In order to handle missing data for PCE and ML models, mean-value imputation and iterative imputation (using multiple imputation by chained equations) were compared^26^. These techniques were chosen to represent the two ends of the spectrum of imputation techniques, with mean-value imputation being the least algorithmically and computationally intensive technique, and iterative imputation being one of the most algorithmically and computationally intensive techniques. Using the full patient cohort, first with PCE variables as inputs and then with PCE+ variables as inputs, imputation and hyperparameter search was carried out using 5-fold cross-validation, and ML model performance was assessed. Supplementary Figure 1 and Supplementary Note 1 provide additional detail on the ML training and cross-validation process.

### Reporting summary

Further information on experimental design is available in the Nature Research Reporting Summary linked to this paper.

## Supplementary information

Supplementary Information

Reporting Summary Checklist

## Data Availability

The datasets analyzed during the current study are not publicly available: due to reasonable privacy and security concerns, the underlying EHR data are not easily redistributable to researchers other than those engaged in the Institutional Review Board-approved research collaborations in the current project. The corresponding author may be contacted for access to EHR data for an IRB approved collaboration.

## Code availability

Code is available at: https://github.com/atward424/ASCVD_ML/.
