# Supplementary material for: Machine learning and atherosclerotic cardiovascular disease risk prediction in a multi-ethnic population
Source: NPJ Digit Med. 2020 Sep 23;3:125. doi: 10.1038/s41746-020-00331-1 (PMC7511400; doi:10.1038/s41746-020-00331-1)

Supplementary Material

Supplementary Data 1. List of all variables in the PCE+ variable set selected by the variable pruning process and used in the final models.

Supplementary Table 1. Level of missingness of PCE variables and additional continuous variables.

Supplementary Table 2. Mean cross-validated and test AUCs for all machine learning models.

Supplementary Table 3. Additional performance metrics for the PCE and GBM models.

Supplementary Table 4. Top 20 Most important features for each model (trained on PCE-eligible patients).

Supplementary Table 5. Mean cross-validated and test AUCs on patients of all races aged 40-79, using iterative imputation and mean-value imputation.

Supplementary Table 6. Test AUCs for ML models trained on non-Hispanic White and African American patients and tested on Asian and Hispanic patients.

Supplementary Table 7. Mean cross-validated and test AUCs on all patients with no missing PCE variables, aged 40–79 years, with oversampling.

Supplementary Table 8. Definitions of cardiovascular diseases.

Supplementary Table 9. Hyperparameters searched for the machine learning models.

Supplementary Note 1. Machine-learning training and cross validation.

Supplementary Figure 1. Explanation of machine learning cross-validation and evaluation.

Supplementary Data 1. List of all variables in the PCE+ variable set selected by the variable pruning process and used in the final models.

PCE variables

- Age
- Female
- HDL cholesterol
- Total cholesterol
- History of Type 2 Diabetes
- Current smoking status
- On antihypertensive medications
- Systolic BP
- Race: African American
- Race: Non-Hispanic White

Clinical variables

- Diastolic BP
- Height
- Weight
- Race: Asian
- Race: Hispanic

Socioeconomic variables

- Median household income
- Percent with up to a 9th grade education
- Percent with up to 12th grade education
- Percent with up to a high school graduation
- Percent with up to some college
- Percent with up to an associate's degree

Percent with up to a bachelor's degree

Utilization variables

Number of other service visits

Number of primary care visits

Number of specialty care visits

Diagnostic variables

CCS: Alcohol-related disorders

CCS: Anxiety disorders

CCS: Aortic; peripheral; and visceral artery aneurysms

CCS: Cancer of head and neck

CCS: Cancer of kidney and renal pelvis

CCS: Chronic kidney disease

CCS: Chronic ulcer of skin

CCS: Deficiency and other anemia

CCS: Delirium, dementia, and amnestic and other cognitive disorders

CCS: Diabetes mellitus with complications

CCS: Diabetes mellitus without complication

CCS: Diseases of white blood cells

CCS: Epilepsy; convulsions

CCS: Essential hypertension

CCS: Heart valve disorders

CCS: HIV infection

CCS: Hypertension with complications and secondary hypertension

CCS: Nervous system congenital anomalies

CCS: Nonspecific chest pain

CCS: Other and ill-defined heart disease

CCS: Other connective tissue disease

CCS: Other endocrine disorders

CCS: Other hematologic conditions

CCS: Other infections; including parasitic

CCS: Other injuries and conditions due to external causes

CCS: Other nervous system disorders

CCS: Other non-traumatic joint disorders

CCS: Pancreatic disorders (not diabetes)

CCS: Parkinson`s disease

CCS: Peri-; endo-; and myocarditis; cardiomyopathy (except that caused by tuberculosis or sexually transmitted disease)

CCS: Septicemia (except in labor)

Family history of emphysema

Lab test variables

Number of abnormal lab test results

Number of normal lab test results

LDL cholesterol

Albumin lab: abnormal

Albumin/Creatinine lab: abnormal

Aspartate aminotransferase lab: normal

Basophils lab: normal

Bilirubin lab: abnormal

Bilirubin lab: normal

C reactive protein lab: abnormal

Calcium lab: normal

Eosinophils lab: abnormal

Eosinophils lab: normal

Fibrinogen lab: abnormal

Glomerular filtration rate lab: abnormal

Glucose lab: abnormal

Lymphocytes lab: abnormal

Monocytes lab: abnormal

Potassium lab: normal

Prostate specific Ag lab: normal

Protein lab: abnormal

Thyroxine lab: abnormal

Urea nitrogen lab: abnormal

Medication variables

Total number of medications prescribed

GPI4: ACE Inhibitors

GPI4: Aminoglycosides

GPI4: Aminopenicillins

GPI4: Angiotensin II Receptor Antagonists

GPI4: Antidementia Agents

GPI4: Antidiarrheal Combinations

GPI4: Antihistamines - Non-Sedating

GPI4: Antihyperlipidemics - Combinations

GPI4: Antipsychotics - Misc.

GPI4: B-Complex w/ C

GPI4: B-Complex w/ Folic Acid

GPI4: Beta Blockers Cardio-Selective

GPI4: Beta-blockers - Ophthalmic

GPI4: Bulk Chemicals - C's

GPI4: Bulk Laxatives

GPI4: Central Muscle Relaxants

GPI4: Cephalosporins - 3rd Generation

GPI4: CMV Agents

GPI4: Cough/Cold/Allergy Combinations

GPI4: Emollient/Keratolytic Agents

GPI4: Emollients

GPI4: Erythromycins

GPI4: H-2 Antagonists

GPI4: HMG CoA Reductase Inhibitors

GPI4: Immunosuppressive Agents

GPI4: Infant Care Products

GPI4: Iron

GPI4: Leprostatics

GPI4: Loop Diuretics

GPI4: Nasal Antiallergy

GPI4: Ophthalmic Immunomodulators

GPI4: Penicillin Combinations

GPI4: Salicylates

GPI4: Surfactant Laxatives

GPI4: Thiazides and Thiazide-Like Diuretics

GPI4: Vitamins w/ Lipotropics

GPI4: Water Soluble Vitamins

Supplementary Table 1. Level of missingness of PCE variables and additional continuous variables.

	Full cohort	PCE cohort
**Variable Name**	**N missing**	**N missing**
**Age**	0	0
**Female**	0	0
**Race**	27,025	11,574
**Systolic BP**	6,698	0
**Diastolic BP**	6,695	0
**On anti-hypertensive medication**	0	0
**HDL cholesterol**	51,802	0
**LDL cholesterol**	52,017	141
**Total cholesterol**	51,499	0
**Current smoker**	1,650	0
**Type 2 diabetes**	0	0
**Weight**	41,521	11,630
**Height**	117,686	51,081
**Median Household Income**	14,144	6,993
**Education Level**	14,132	6,988

Abbreviations: PCE–pooled cohort equation; BP – blood pressure; HDL – high density lipoprotein; LDL – density lipoprotein

Supplementary Table 2. Mean cross-validated and test AUCs for all machine learning models.

				Cross- Validated AUCs						Held-Out Test AUCs					
Race	Age	Missing data	Variables	LR	Lasso	RF	GBM	XGBoost	PCE	LR	Lasso	RF	GBM	XGBoost	PCE
Black/NHW	40-79	No	PCE	0.758	0.757	0.757	0.759	0.757	0.747	0.770	0.769	0.767	0.772	0.772	0.764
Black/NHW	40-79	No	PCE+	0.685	0.762	0.755	0.759	0.758	0.747	0.699	0.779	0.777	0.781	0.782	0.764
Black/NHW	40-79	Yes	PCE	0.762	0.762	0.764	0.765	0.763	NA	0.773	0.773	0.779	0.778	0.777	NA
Black/NHW	40-79	Yes	PCE+	0.685	0.761	0.754	0.758	0.758	NA	0.699	0.779	0.776	0.778	0.782	NA
Asian	40-79	No	PCE	0.767	0.769	0.760	0.767	0.765	0.761	0.802	0.804	0.756	0.777	0.770	0.807
Asian	40-79	No	PCE+	0.705	0.753	0.774	0.773	0.765	0.761	0.727	0.803	0.768	0.781	0.795	0.807
Asian	40-79	Yes	PCE	0.778	0.780	0.781	0.780	0.779	NA	0.797	0.798	0.759	0.769	0.772	NA
Asian	40-79	Yes	PCE+	0.676	0.752	0.786	0.784	0.765	NA	0.687	0.803	0.782	0.785	0.795	NA
Hispanic	40-79	No	PCE	0.742	0.757	0.752	0.758	0.754	0.761	0.748	0.752	0.728	0.724	0.729	0.742
Hispanic	40-79	No	PCE+	0.700	0.746	0.761	0.764	0.771	0.761	0.716	0.768	0.700	0.698	0.694	0.742
Hispanic	40-79	Yes	PCE	0.752	0.763	0.738	0.757	0.751	NA	0.792	0.795	0.788	0.780	0.784	NA
Hispanic	40-79	Yes	PCE+	0.700	0.747	0.767	0.772	0.771	NA	0.700	0.769	0.753	0.763	0.694	NA
All	40-79	No	PCE	0.763	0.763	0.765	0.765	0.765	0.758	0.776	0.776	0.772	0.773	0.774	0.775
All	40-79	No	PCE+	0.706	0.768	0.766	0.770	0.769	0.758	0.749	0.784	0.773	0.779	0.784	0.775
All	40-79	Yes	PCE	0.769	0.769	0.770	0.771	0.771	NA	0.779	0.779	0.782	0.784	0.783	0.774*
All	40-79	Yes	PCE+	0.728	0.768	0.775	0.777	0.769	NA	0.751	0.784	0.785	0.790	0.784	0.774*
All	18+	No	PCE	0.818	0.818	0.816	0.818	0.819	0.812	0.821	0.821	0.814	0.819	0.823	0.817
All	18+	No	PCE+	0.799	0.821	0.817	0.821	0.822	0.812	0.801	0.826	0.818	0.824	0.830	0.817
All	18+	Yes	PCE	0.830	0.830	0.830	0.830	0.831	NA	0.831	0.831	0.831	0.831	0.836	NA
All	18+	Yes	PCE+	0.812	0.821	0.831	0.834	0.822	NA	0.808	0.825	0.831	0.835	0.830	NA

Supplementary Table 3. Additional performance metrics on the held-out test set for the PCE and GBM models.

	PCE: PCE Cohort (N=131,721)				GBM*: PCE Cohort (N=131,721)				GBM*: Full Cohort (N=262,923)			
Percentage of patients in "high-risk" category	Sensitivity	Specificity	Precision	F1 Score**	Sensitivity	Specificity	Precision	F1 Score**	Sensitivity	Specificity	Precision	F1 Score**
1	0.069	0.991	0.110	0.536	0.074	0.991	0.119	0.540	0.094	0.991	0.146	0.551
2	0.135	0.982	0.107	0.552	0.140	0.982	0.112	0.554	0.196	0.983	0.152	0.578
5	0.275	0.954	0.087	0.552	0.292	0.954	0.093	0.556	0.377	0.955	0.117	0.575
10	0.404	0.905	0.064	0.528	0.424	0.905	0.067	0.531	0.559	0.907	0.087	0.549
25	0.662	0.757	0.042	0.469	0.662	0.757	0.042	0.469	0.764	0.758	0.047	0.475
50	0.860	0.506	0.027	0.362	0.842	0.506	0.027	0.361	0.900	0.506	0.028	0.363
75	0.948	0.253	0.020	0.222	0.940	0.253	0.020	0.221	0.969	0.253	0.020	0.222
90	0.986	0.101	0.017	0.109	0.989	0.101	0.017	0.109	0.988	0.101	0.017	0.109
95	0.997	0.051	0.017	0.065	0.997	0.051	0.017	0.065	0.994	0.051	0.016	0.064
98	1.000	0.020	0.016	0.036	1.000	0.020	0.016	0.036	0.999	0.020	0.016	0.035
99	1.000	0.010	0.016	0.026	1.000	0.010	0.016	0.026	1.000	0.010	0.016	0.025

*GBM models presented here were trained on all variables and used iterative imputation to fill in missing variables.

**The F1 Score was weighted to account for class imbalance.

Abbreviations: AUC–area under receiver operating characteristic curve; PCE–pooled cohort equation; PCE+–pooled cohort equation variables with additional electronic health record variables; GBM–gradient boosting machine; CI-confidence interval

Supplementary Table 4. Top 20 most important features for each model (trained on PCE-eligible patients)

Rank of variable importance (1=top)	LRL2 (111 variables used)	LRLasso (91 variables used)	RF (108 variables used)	GBM (108 variables used)	XGBoost (56 variables used)
1	Age	Age	Age	Age	Age
2	height	Number of normal lab test results	Systolic BP	Systolic BP	Female
3	HDL cholesterol	Systolic BP	Total cholesterol	LDL cholesterol	Systolic BP
4	Diastolic BP	History of Type 2 Diabetes	CCS: Essential hypertension	Weight	History of Type 2 Diabetes
5	Median household income	Female	LDL cholesterol	Total cholesterol	CCS: Essential hypertension
6	Systolic BP	HDL cholesterol	Weight	HDL cholesterol	C reactive protein lab: abnormal
7	Weight	Current smoker	HDL cholesterol	Diastolic BP	Current smoker
8	Number of normal lab test results	Total medications prescribed	height	CCS: Essential hypertension	On antihypertensive medications
9	Number of abnormal lab test results	Bilirubin lab: normal	Diastolic BP	height	Race: African American

10	Total medications prescribed	Total cholesterol	Median household income	Median household income	GPI4: Thiazides and Thiazide-Like Diuretics
11	Number of specialty care visits	Diastolic BP	On antihypertensive medications	On antihypertensive medications	Albumin/Creatinine lab: abnormal
12	Total cholesterol	Percent with up to a bachelor's degree	Percent with up to a bachelor's degree	Percent with up to an associate's degree	GPI4: Beta Blockers Cardio-Selective
13	LDL cholesterol	Basophils lab: normal	Percent with up to an associate's degree	Total medications prescribed	HDL cholesterol
14	Percent with up to a bachelor's degree	CCS: Essential hypertension	Percent with up to some college	Percent with up to a bachelor's degree	Percent with up to a high school graduation
15	Number of primary care visits	height	Percent with up to 12th grade education	Percent with up to some college	Weight
16	Female	GPI4: Thiazides and Thiazide-Like Diuretics	Percent with up to a high school graduation	Percent with up to 12th grade education	CCS: Diabetes mellitus with complications
17	Percent with up to an associate's degree	Number of abnormal lab test results	Total medications prescribed	Percent with up to a high school graduation	LDL cholesterol
18	On antihypertensive medications	Albumin/Creatinine lab: abnormal	Percent with up to a 9th grade education	History of Type 2 Diabetes	Eosinophils lab: abnormal

19	CCS: Essential hypertension	GPI4: Antihistamines - Non-Sedating	Number of abnormal lab test results	Number of abnormal lab test results	Diastolic BP
20	Bilirubin lab: normal	Percent with up to an associate's degree	Number of primary care visits	Percent with up to a 9th grade education	Percent with up to some college

Percent refers to percent of census block in all instances.

Abbreviations: PCE–pooled cohort equation; LRL2–logistic regression with an L_2 penalty; LRLasso–logistic regression with an L_1 (lasso) penalty; RF–random forest; GBM–gradient boosting machine; XGBoost–extreme gradient boosting; BP–blood pressure; HDL–high-density lipoprotein; LDL–low-density lipoprotein; CCS–clinical classification software; GPI4–first four digits of generic product identifier

Supplementary Table 5. Mean cross-validated and test AUCs on patients of all races aged 40-79, using iterative imputation and mean-value imputation.

	Mean cross-Validated AUCs - Iterative Imputer			Mean cross-Validated AUCs - Mean Imputer			Test AUCs (95% CI)			
**Variables**	**LRL2**	**GBM**	**RF**	**LRL2**	**GBM**	**RF**	**GBM (iterative)**	**GBM (mean-value)**	**PCE (iterative)**	**PCE (mean-value)**
**PCE**	0.781	0.784	0.784	0.753	0.768	0.768	0.784 (0.768-0.800)	0.752 (0.736-0.768)	0.774 (0.757-0.790)	0.773 (0.756-0.790)
**PCE+**	0.728	0.792	0.788	0.728	0.776	0.773	0.790 (0.774-0.805)	0.745 (0.729-0.761)	0.774 (0.757-0.790)	0.772 (0.755-0.788)

Abbreviations: AUC–area under receiver operating characteristic curve; PCE–pooled cohort equation; PCE+–pooled cohort equation variables with additional electronic health record variables; LRL2–logistic regression with an L_2 penalty; RF–random forest; GBM–gradient boosting machine; CI-confidence interval

Imputation was done within the cross-validation loop; for each fold, only the training folds were used to train the imputer, then the missing values of the held-out validation fold were imputed using the trained imputer. For the test set, the imputers were retrained on all of the training data, then used to impute missing variables in the test data.

Supplementary Table 6: Test AUCs for ML models trained on non-Hispanic White and African American patients and tested on Asian and Hispanic patients.

	Test AUC (95% CI)	
**Model**	**Asian**	**Hispanic**
LRL2	0.715 (0.666-0.763)	0.696 (0.611-0.781)
GBM	0.797 (0.760-0.835)	0.726 (0.646-0.805)
RF	0.794 (0.756-0.832)	0.730 (0.651-0.808)
LRLasso	0.797 (0.759-0.835)	0.758 (0.685-0.831)
XGBoost	0.780 (0.740-0.820)	0.725 (0.645-0.804)
PCE	0.807 (0.771-0.843)	0.742 (0.666-0.818)

Abbreviations: AUC–area under receiver operating characteristic curve; PCE–pooled cohort equation; LRL2–logistic regression with an L_2 penalty; RF–random forest; GBM–gradient boosting machine; XGBoost – extreme gradient boosting; LRLasso–logistic regression with an L1 (lasso) penalty; CI-confidence interval

Supplementary Table 7. Mean cross-validated and test AUCs on all patients with no missing PCE variables, aged 40–79 years, with oversampling.

		Mean cross-Validated AUCs		Test AUCs	
**Variables**	**Oversample Rate**	**GBM**	**PCE**	**GBM**	**PCE**
**PCE**	**x1**	**0.765**	**0.758**	**0.775**	**0.771**
**PCE**	**x2**	**0.764**	**0.758**	**0.775**	**0.771**
**PCE**	**x5**	**0.765**	**0.758**	**0.776**	**0.771**
**PCE**	**x10**	**0.765**	**0.758**	**0.775**	**0.771**
**PCE+**	**x1**	**0.77**	**0.758**	**0.757**	**0.771**
**PCE+**	**x2**	**0.769**	**0.758**	**0.772**	**0.771**
**PCE+**	**x5**	**0.77**	**0.758**	**0.769**	**0.771**
**PCE+**	**x10**	**0.769**	**0.758**	**0.774**	**0.771**

Abbreviations: AUC–area under receiver operating characteristic curve; PCE–pooled cohort equation; PCE+–pooled cohort equation variables with additional electronic health record variables; GBM–gradient boosting machine. Because ASCVD events are relatively rare, we assessed the usefulness of oversampling patients with ASCVD using cross-validation. Oversampling was done within the cross-validation loop; that is, for each fold, only the patients in the training folds, not in the validation fold, were oversampled. The validation fold retained the same number of patients with ASCVD so as to give a true estimate of the AUC. Oversampling did not improve performan

Supplementary Table 8. Definitions of cardiovascular diseases.

	ICD-9-CM	ICD-10-CM
Myocardial infarction	410.*	I21.*, I22.*, I23.3, I24.0, I24.9, I25.9, I51.3,
Coronary artery disease	411.*, 413.*, 414.*	I20.*, I23.7, I24.*, I25.*, T82.85,
Ischemic stroke	433.01, 433.11, 433.21, 433.31, 434.81, 433.91, 434.11, 434.91, 436.*	G46.*, I63.*, I67.85, I69.30, I77.89, P91.0, Z86.73
Hemorrhagic stroke	430.*, 431.*, 432.*	I60.*, I61.*, I62.*, I63.89, I67.1, P52.*, P54.8, S06.2X, S06.4X, S06.5X
Atrial fibrillation, heart failure	427.31, 428.*	I27.29, I48.0, I48.1, I48.2, I48.91, I50.*, I51.9, Z86.79
Other cardiovascular disease	412.*, 346.6* 433.*, 434.*, 435.*, 437.*, 438.*, 440.*, V45.81, or V45.82	G43.6*, G45.*, G46.*, G81.94, G83.9, G93.89, G93.9, G95.19, I25.1, I25.2, I63.*, I65.*, I66.*, I67.*, I68.*, I69.*, I70.*, I72.5, I76, I77.75, I77.89, I77.9, I99.8, M47.01, P91.0, Z98.61

Abbreviations: ICD-9-CM–International classification of diseases, 9th revision; ICD-10-CM–International classification of diseases, 10th revisio

Supplementary Table 9. Hyperparameters searched for the machine learning models.

Model	Hyperparameter	Values
LRL2	C	0.01, 0.03, 0.1, 0.3, 1, 3, 10
LRLasso	C	0.001, 0.01, 0.1, 1
RF	n_estimators	100, 200, 400, 500, 600, 1000, 2000, 4000
	max_depth	4,6,7,8,9,10, None
	min_samples_split	1,5,10
	max_features	0.25, 0.35, 0.5, 0.75, 1
GBM	n_estimators	100, 200, 400, 500, 600, 800, 1000, 2000, 4000
	max_depth	2, 3, 4, 5, 6, 7, 8, 9
	subsample	0.25, 0.5, 0.75, 1
	max_features	sqrt, log2, 0.25, 0.35, 0.5, 0.75, 1
	learning_rate	0.001, 0.01, 0.03, 0.1
XGBoost	n_estimators	100, 200, 400, 500, 600, 800, 1000, 2000, 4000
	max_depth	2, 3, 4, 5, 6, 7, 8, 9

	learning_rate	0.001, 0.01, 0.03, 0.1
	reg_alpha	0,1,10
	reg_lambda	0.1,1,10

Abbreviations: LRL2–logistic regression with an L_2 penalty; LRLasso–logistic regression with an L_1 (lasso) penalty; RF–random forest; GBM–gradient boosting machine; XGBoost–extreme gradient

Supplementary Note 1.

This note provides additional details regarding machine learning training, cross-validation, and test approach.

First, the full dataset was randomly split by patient into an 80% training/validation set, and a 20% held-out test set. This 80/20 split is a standard convention, and since our dataset is large, we expect the 20% held-out test set to be a representative sample of the full dataset. This held-out test set was only defined once, at the beginning of the study.

The following steps were repeated twice, with appropriate modifications: first, using only variables which are used as inputs to the PCE, and second, using the additional variables extracted from the EHR.

Next, we used 5-fold cross-validation to tune hyperparameters using all of the variables extracted from the EHR. For this first hyperparameter tuning phase, we used a grid-search approach with a restricted set of the hyperparameter values shown in Supplementary Table 9. Additional details of the roles of individual hyperparameters can be found at https://scikit-learn.org/0.21/. We fixed the cross-validation folds a priori, so each ML model was trained and validated on the same sets of data for each fold. For each algorithm (Logistic Regression, Lasso, Random Forest, Gradient Boosting Machine, Extreme Gradient Boosting), the model with the highest average AUC across the 5 folds was selected from the hyperparameter grid search. For this phase, simple imputation was used to fill in missing variables.

We then used the best RF, GBM, XGBoost, and LRLasso model for feature selection, as the logistic regression model had poor cross-validated performance. This step was not needed for the analysis which only considered the PCE variables. We defined a composite score by examining the default feature rankings from each model. For RF, GBM, and XGBoost, the mean decrease in impurity (MDI) was used to assess feature importance. Although the MDI has some shortcomings regarding variable types (binary, categorical, and continuous) and missingness levels, we were primarily concerned with removing binary variables. For the LRLasso model, a feature's importance was determined by multiplying the absolute value of the coefficient by the standard deviation of the variable. For each of the four models, the most important variable was assigned a rank of 1, the second most important variable was assigned "2", etc. The composite score for each variable was defined as the minimum rank across the four models. Any feature which had a composite score of greater than 100 was excluded from further analysis; a second round of model training was done and composite scores were recalculated, and any feature which had a composite score of greater than 50 was excluded from further analysis.

With the restricted variable set, we then performed a broader hyperparameter grid search for each algorithm, using all of the parameter values in Supplementary Table 9, again using the pre-selected cross-validation folds within the training set. In addition to these hyperparameters, we also tested whether iterative imputation or mean value imputation resulted in a higher cross-validated AUC. The imputation was done within the cross-validation folds; in other words, a separate imputer was trained on each of the training CV folds ("training" the mean value imputer simply consisted of calculating the mean for each variable within that fold) and then run on the held-out CV. Boolean missing flag variables were added for each variable that had missing data.

Once the best imputer was chosen and the new hyperparameters were set for each model, oversampling of the minority class (patients who developed ASCVD in the 5-year follow-up period) was tested at different rates. This consisted of randomly resampling individuals in the minority class, with replacement, until a desired number of observations with the outcome were included in the dataset. The numbers tested were 2x, 5x, and 10x the minority class size. Oversampling was only done on the training folds, not on the evaluation folds, so that the cross-validated AUCs are comparable across all oversampling rates.

After all these steps were completed, the best-performing pipeline (consisting of the best imputer, the best oversampling rate, and the best model hyperparameters) was retrained on the entire training dataset. This fully trained pipeline was then used to predict outcomes on the held-out test set. We report held-out test set results, including AUC, sensitivity, specificity, precision, and F1-score weighted for class imbalance (the F1 score for the majority class and minority class were calculated separately, and we report the average).

Once this process was completed for the entire cohort, we added an extra round of hyperparameter tuning on several sub-cohorts: PCE-eligible patients, all patients aged 40-79 (including those with missing or out-of-range PCE variables), Hispanic patients, Asian patients, and Non-Hispanic White (NHW) and African American (AA) patients (considered together). This hyperparameter tuning was again done using the predefined 5 cross validation folds (which weren't equally sized for some of these sub-cohorts), and the final metrics are reported on the corresponding patients in the held-out test set. For patient populations which had no missing data (e.g. PCE-eligible patients), imputation was not needed, and the boolean missing flag variables were not created.

As a comparison to the PCE, which was primarily validated in NHW and AA patients, we also used the models trained only on NHW and AA patients to predict outcomes for Hispanic and Asian patients. Results are shown in Supplementary Table 6.

Supplementary Figure 1. Explanation of machine learning cross-validation and evaluation.

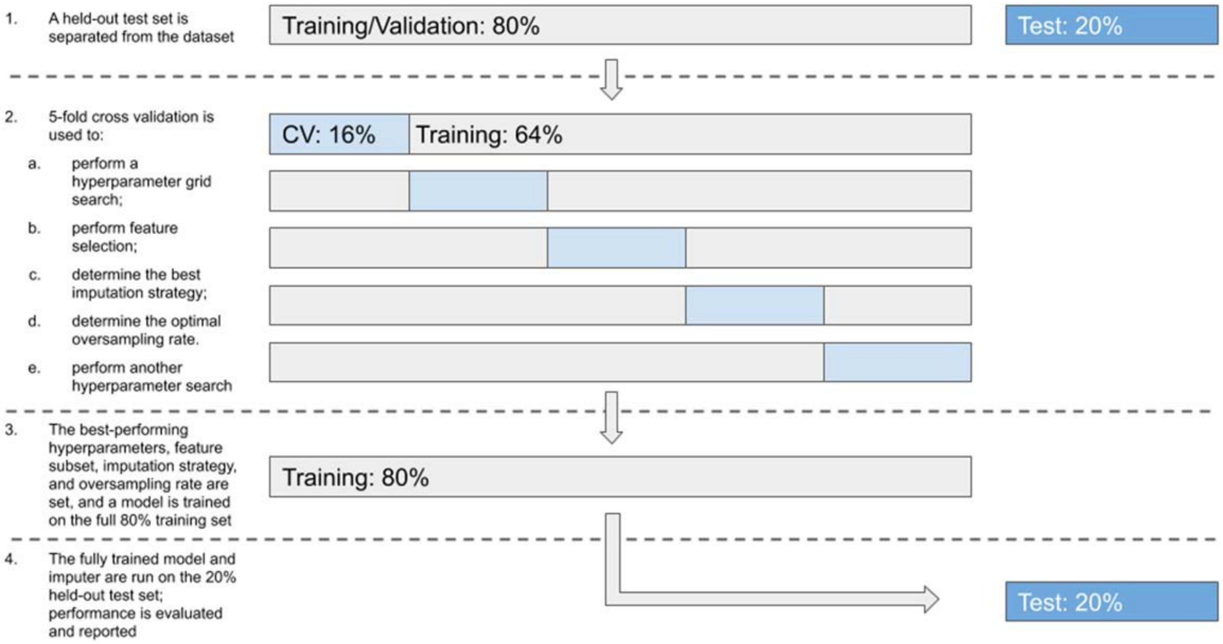

Supplement: Supplementary file 1 — Supplementary Information [file 41746_2020_331_MOESM1_ESM.pdf]
