# Supplementary material for: Machine learning and atherosclerotic cardiovascular disease risk prediction in a multi-ethnic population
Source: NPJ Digit Med. 2020 Sep 23;3:125. doi: 10.1038/s41746-020-00331-1 (PMC7511400; doi:10.1038/s41746-020-00331-1)
Supplement: Supplementary file 2 — Reporting Summary Checklist [file 41746_2020_331_MOESM2_ESM.pdf]

nature research

Corresponding author(s): Fatima Rodriguez

Last updated by author(s): Aug 26, 2020

Reporting Summary

Nature Research wishes to improve the reproducibility of the work that we publish. This form provides structure for consistency and transparency in reporting. For further information on Nature Research policies, see our Editorial Policies and the Editorial Policy Checklist.

Statistics

For all statistical analyses, confirm that the following items are present in the figure legend, table legend, main text, or Methods section.

n/a	Confirmed	
☐	☒	The exact sample size (*n*) for each experimental group/condition, given as a discrete number and unit of measurement
☐	☒	A statement on whether measurements were taken from distinct samples or whether the same sample was measured repeatedly
☐	☒	The statistical test(s) used AND whether they are one- or two-sided<br>*Only common tests should be described solely by name; describe more complex techniques in the Methods section.*
☒	☐	A description of all covariates tested
☒	☐	A description of any assumptions or corrections, such as tests of normality and adjustment for multiple comparisons
☐	☒	A full description of the statistical parameters including central tendency (e.g. means) or other basic estimates (e.g. regression coefficient) AND variation (e.g. standard deviation) or associated estimates of uncertainty (e.g. confidence intervals)
☒	☐	For null hypothesis testing, the test statistic (e.g. *F*, *t*, *r*) with confidence intervals, effect sizes, degrees of freedom and *P* value noted<br>*Give P values as exact values whenever suitable.*
☒	☐	For Bayesian analysis, information on the choice of priors and Markov chain Monte Carlo settings
☒	☐	For hierarchical and complex designs, identification of the appropriate level for tests and full reporting of outcomes
☒	☐	Estimates of effect sizes (e.g. Cohen's *d*, Pearson's *r*), indicating how they were calculated

Our web collection on statistics for biologists contains articles on many of the points above.

Software and code

Policy information about availability of computer code

Data collection	For data collection, we used the EPIC electronic health record (EHR) provided to the Virtual Data Warehouse (VDW) which was developed with NIH support and is a standardized method for sharing EHR information using standard variables, variable definitions, and formats.
Data analysis	Analysis was performed in Python 3.7 using the scikit-learn and xgboost packages, versions 0.21.2 and 0.90, respectively.

For manuscripts utilizing custom algorithms or software that are central to the research but not yet described in published literature, software must be made available to editors and reviewers. We strongly encourage code deposition in a community repository (e.g. GitHub). See the Nature Research guidelines for submitting code & software for further information.

Data

Policy information about availability of data

All manuscripts must include a data availability statement. This statement should provide the following information, where applicable:

- Accession codes, unique identifiers, or web links for publicly available datasets
- A list of figures that have associated raw data
- A description of any restrictions on data availability

The datasets analyzed during the current study are not publicly available: due to reasonable privacy and security concerns, the underlying EHR data are not easily redistributable to researchers other than those engaged in the Institutional Review Board-approved research collaborations in the current project. The corresponding author may be contacted for access to EHR data for an IRB approved collaboration.

Code Availability
Code is available at: https://github.com/atward424/ASCVD_ML/

Field-specific reporting

Please select the one below that is the best fit for your research. If you are not sure, read the appropriate sections before making your selection.

☒ Life sciences ☐ Behavioural & social sciences ☐ Ecological, evolutionary & environmental sciences

For a reference copy of the document with all sections, see nature.com/documents/nr-reporting-summary-flat.pdf

Life sciences study design

All studies must disclose on these points even when the disclosure is negative.

Sample size	Sample size was determined by including all patients that met inclusion criteria for ML training and testing for ASCVD risk prediction: Adults with no prior CVD and not on statins at baseline, who were over 18 years of age in a community-based outpatient healthcare system in Northern California between January 1, 2009 and December 31, 2018 with at least two outpatient visits that were at least 1 year apart, and at least 5 years of follow-up if there were no outcome events.
Data exclusions	There were no additional exclusions beyond the inclusion criteria as above.
Replication	ML model performance was assessed on an unseen held-out test set to assess performance. Assessing performance in different patient cohorts may be the basis of future studies.
Randomization	As this was a study to train and test ML model performance on a large patient cohort (using an 80%/20% split stratified by outcome), there was no randomization to experimental/control groups.
Blinding	There was no experimental versus control group allocation based on the study design. All patients were included in a single cohort that was split into training and test sets as above.

Reporting for specific materials, systems and methods

We require information from authors about some types of materials, experimental systems and methods used in many studies. Here, indicate whether each material, system or method listed is relevant to your study. If you are not sure if a list item applies to your research, read the appropriate section before selecting a response.

Materials & experimental systems

n/a	Involved in the study	
☒	☐	Antibodies
☒	☐	Eukaryotic cell lines
☒	☐	Palaeontology and archaeology
☒	☐	Animals and other organisms
☒	☐	Human research participants
☒	☐	Clinical data
☒	☐	Dual use research of concern

Methods

n/a	Involved in the study	
☒	☐	ChIP-seq
☒	☐	Flow cytometry
☒	☐	MRI-based neuroimaging
